# Fermented foods, their microbiome and its potential in boosting human health

**DOI:** 10.1111/1751-7915.14428

**Published:** 2024-02-23

**Authors:** Vincenzo Valentino, Raffaele Magliulo, Dominic Farsi, Paul D. Cotter, Orla O'Sullivan, Danilo Ercolini, Francesca De Filippis

**Affiliations:** ^1^ Department of Agricultural Sciences University of Naples Federico II Portici Italy; ^2^ NBFC‐National Biodiversity Future Center Palermo Italy; ^3^ Department of Food Biosciences Teagasc Food Research Centre Moorepark Fermoy Ireland; ^4^ APC Microbiome Ireland National University of Ireland Cork Ireland; ^5^ VistaMilk, Fermoy Cork Ireland; ^6^ Task Force on Microbiome Studies University of Naples Federico II Portici Italy

## Abstract

Fermented foods (FFs) are part of the cultural heritage of several populations, and their production dates back 8000 years. Over the last ~150 years, the microbial consortia of many of the most widespread FFs have been characterised, leading in some instances to the standardisation of their production. Nevertheless, limited knowledge exists about the microbial communities of local and traditional FFs and their possible effects on human health. Recent findings suggest they might be a valuable source of novel probiotic strains, enriched in nutrients and highly sustainable for the environment. Despite the increasing number of observational studies and randomised controlled trials, it still remains unclear whether and how regular FF consumption is linked with health outcomes and enrichment of the gut microbiome in health‐associated species. This review aims to sum up the knowledge about traditional FFs and their associated microbiomes, outlining the role of fermentation with respect to boosting nutritional profiles and attempting to establish a link between FF consumption and health‐beneficial outcomes.

## Graphical abstract

Mechanism for γ‐PGA electrofermentation (left); charge output and γ‐PGA production in electrofermentation (center, top); SEM of γ‐PGA‐containing EPS on carbon fiber in electrofermentation and conventional ferementation (center, bottom); (^1^H)‐NMR of γ‐PGA‐enriched EPS in electrofermentation (right).

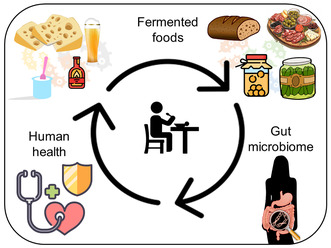

## INTRODUCTION

For centuries, food fermentation has been used to preserve foods and ameliorate their sensorial quality, safety and shelf‐life (Marsh et al., 2014). The first evidence of fermentation dates back 8000 years to cheese production (Ross et al., 2002), although recent evidence suggests that fermented foods (FFs) could have been consumed at the dawn of human evolution (Amato et al., 2021).

Since then, the mode of FF production has greatly evolved, owing to developments in technology, with the food industry moving from spontaneous to well‐controlled and even precision fermentations (Chai et al., 2022). Advances in research methods, including improvements in the capacity to isolate of microbial strains and the widespread use of “omics sciences to explore the genetic profiles the microbiomes”, have led to the selection of well‐characterised strains to be used as starter cultures, providing highly standardised FFs on a global scale.

However, in parallel to the industrialisation of practices associated with the production of some specific FFs, it is estimated that a broader range of ~5000 varieties of, primarily artisanally produced, FFs might be consumed nowadays worldwide (Tamang et al., 2020). These traditional FFs are predominantly embedded within local population cultures, harbouring a plethora of microbial species and strains that might be valuable after ad hoc screening and selection of their technologic and probiotic properties. Further research efforts are needed to better describe the microbial diversity within traditional FFs and depict the metabolic activities exerted by product‐specific microbiome.

Therefore, the purpose of this review is to summarise the knowledge about FFs and their associated microbiome, highlighting the relevance of food fermentation for humans, focusing on its technological and nutritional outcomes, as well as on the beneficial effects of FFs. Furthermore, we describe the microbial communities residing in traditional FFs worldwide, focusing on the role of each microbial group in the production and stability of the final product.

## FERMENTATION AS A SUSTAINABLE TOOL TO ENSURE QUALITY AND SAFETY OF PERISHABLE PRODUCTS

According to the International Scientific Association for Probiotics and Prebiotics (ISAPP), FFs are “food made through desired microbial growth and enzymatic conversions of food components.” The fermentation process is among the most effective ways for extending raw material shelf‐life, especially for world areas with limited access to electricity (Marco et al., 2021). The utility of fermentation as a means food preservation is due to the biosynthesis of organic acids, alcohols, bacteriocins and other antimicrobials as a result of metabolism by the FF microbiome (Ross et al., 2002). Thus, during fermentation, multiple biochemical and physical modifications of food components occur, resulting in the improvement of food safety and shelf‐life. For example, during lactic acid fermentation, bacteria use six‐carbon mono‐ or oligosaccharides as carbon sources and mainly produce lactic acid (Amit et al., 2017), which leads to a drop in the pH (Zapaśnik et al., 2022), thus inhibiting several spoilage and pathogenic species that might be present in the raw materials. Besides this, alcoholic fermentation consists of the anaerobic conversion of sugars into ethanol and carbon dioxide (Buglass, 2011). High concentrations of ethanol might lead to cell death through disruption of membrane integrity, as well as altering the biosynthesis of essential components such as fatty acids, lipids and outer membranes (Liu & Qureshi, 2009). In some settings, alcoholic is followed by acetic fermentation, which involves the aerobic oxidation of ethanol to acetic acid (Gomes et al., 2018). This fermentation is the pivotal step in the production of vinegar and kombucha, albeit being undesirable in the production of wine and beer. Even though lactic and alcoholic are the most studied and employed form of food fermentation, a number of other fermentation types exist, for example, the malolactic and the alkaline fermentations. The former is relevant in wine ageing and consists of the conversion of malic acid (present in grapes) into lactic acid and carbon dioxide, with considerable consequences on sensory profile and long‐term stability of wines (Paramithiotis et al., 2022), whereas the latter involves protein hydrolysis with the release of ammonia, typically occurring in traditional high‐pH FFs from Africa and Asia such as *nattō* (Owusu‐Kwarteng et al., 2022). A plethora of microorganisms can be involved in food fermentation. Generally, Lactic Acid Bacteria (LAB) perform lactic fermentation, whereas yeasts and a few bacteria produce ethanol from sugar metabolism (Malakar et al., 2020). Furthermore, Acetic Acid Bacteria (AAB), a group comprising 19 genera from the *Acetobacteriaceae* family, can perform the acetic fermentation (Gomes et al., 2018; Table 1). During fermentation, the accumulation of metabolites, such as ethanol and acids, is not the sole mechanism protecting foods from harmful microbes. On the contrary, other beneficial compounds produced by fermenting microbes, or even their sole presence, might preserve food matrices. For example, several LAB species are known to excrete bacteriocins, short peptides with antimicrobial activity (De Filippis et al., 2020; Mokoena, 2017). Several studies have shown that bacteriocins are being active against foodborne pathogens, both in vitro and in situ. For example, Ye et al. (2021) recently characterised a novel bacteriocin produced by a *Lacticaseibacillus paracasei* (formerly *Lactobacillus paracasei*; Zheng et al., 2020) strain which is active against the foodborne pathogens *Listeria monocytogenes* and *Salmonella typhimurium*, while Martinez et al. (2016) assessed the inhibitory effect of nisin on the growth of *Listeria monocytogenes* and germination of *Bacillus cereus* spores in milk. Interestingly, several FFs are sources of strains belonging to species that have been shown to be bacteriocin‐producing. Indeed, the FFs' milk kefir (Petrova et al., 2021) and table olives (Hurtado et al., 2012) are frequently reported to be a source of bacteriocin‐producing LAB. This is also true of cheeses, with soft cheese harbouring greater amounts compared to hard and semihard cheeses (Trejo‐González et al., 2022). Besides bacteriocin production, food stability is further improved during fermentation due to the fact that technologically active bacteria frequently outcompete potential pathogenic and spoilage taxa (Rul & Monnet, 2015). Indeed, LAB can inhibit the growth of pathogens in FFs by competing for nutrients (Ibrahim et al., 2021; Vieco‐Saiz et al., 2019). For example, Martín et al. (2022) showed that the addition of strains of *Lacticaseibacillus casei* and *Lactococcus garvieae* during the production of a Spanish cheese effectively inhibited the growth of *Listeria monocytogenes*, while Siedler et al. (2020) showed that LAB species may counteract the growth of pathogens and spoilage microbes by depriving them of manganese.

**TABLE 1:** Overview of the most common food fermentations.

Fermentation	Main taxa involved	Main products	Main FFs produced
Lactic	*Lactobacillaceae*, *Leuconostocaceae*, *Streptococcaceae*	Lactic acid (homolactic), CO_2_, ethanol (heterolactic)	Dairy (yogurt, cheeses, kefir), sauerkraut, kimchi, pickles, tempeh, fermented meats
Alcoholic	*Saccharomyces* spp., *Kloeckera* spp.	Ethanol, CO_2_	Wine, beer, kefir
Acetic	*Acetobacter* spp., *Gluconacetobacter*, *Gluconobacter*	Acetate, EPS	Chocolate, coffee, vinegar, specialty beers, water kefir
Propionic	*Propionibacterium* spp.	Propionate, acetate, CO_2_, succinate (Wood–Werkman pathway)	Swiss‐type cheeses

In some FFs, microbial fermentation of the raw materials is necessary to make them edible. For example, multiple fermentations by a mix of yeasts, AAB and LAB are necessary to digest the pulp covering the cocoa beans and develop chocolate flavour precursors and antioxidants that would otherwise be absent (Goya et al., 2022; Rahardjo et al., 2022).

## INNOVATIONS IN FOOD FERMENTATION TO IMPROVE GLOBAL HEALTH

Besides being a tool for improving the shelf‐life of foods, making them edible or safer for human consumption, fermentation can be considered one of the solutions to reduce the dramatically high environmental impact of the food industry. Indeed, fermentation is widely recognised as a sustainable process, with very low gas emission and a high production yield (Rastogi et al., 2022) and is a possible route to produce nutrient foods from alternative sources (e.g., legumes and vegetables), potentially offering alternatives to less sustainable foods. For instance, fermentation of protein‐rich foods such as legumes with LAB strains increases the concentration of bioactive compounds (Kårlund et al., 2020) and might ameliorate the amino acid profiles (Emkani et al., 2022), thus providing a sustainable and nutritionally valid alternative protein source compared to meats, as well as helping in fighting hunger and malnutrition in low‐income countries.

FFs have been a consistent part of human diets across various cultures for thousands of years, and different FFs and beverages are widespread across the world (Tamang et al., 2020). However, the production and consumption of FFs traditionally consumed only in specific countries are changing with globalisation (Rastogi et al., 2022). For instance, products obtained from fermented legumes (e.g., tempeh) are traditionally consumed in several Asiatic countries and are a consistent part of their cultural heritage, but have attracted greater interest from Western populations quite recently (Vinderola et al., 2023). While some of these FFs have continued to be produced using traditional methods of production, that is, depending on local resources and environmental conditions, most have undergone significant technological advancements in order to meet global demand (Panda & Shetty, 2018). Despite the limited scientific understanding of fermentation and microorganisms in the past, microbial communities in small‐scale fermentations using traditional methods have been identified (Holzapfel, 2002; Panda & Shetty, 2018). However, the challenge for scientists lies in managing large scale production without compromising the distinctive characteristics of the traditional products. Therefore, the characterisation of traditional fermentations followed by their optimisation and application on a large‐scale might represent a promising starting point towards a more sustainable food system, providing nutrient‐rich products (see Section: “Fermentation boosts the nutritional value of foods”) with limited waste produced. The need for novel sustainable food production strategies is pushing the food industry to explore paths for innovation in food fermentation. Whole‐genome sequencing and metabolic network reconstruction can help to identify safe and productive microbial strains prior to any in vitro test, overcoming the “trial and error” approach and leading us towards precision fermentation. These technologies open large‐scale frontiers, such as the production of nutrients and the development of appealing novel foods from previous waste streams (Jahn et al., 2023). Indeed, several companies are testing the production of food products with complete amino acid profiles and acceptable sensorial characteristics from microbial biomass, to use them for the production of surrogate meat (Humpenöder et al., 2022; Wackett, 2020) and dairy products (Linder, 2019). Therefore, revalorisation of agricultural wastes through cost‐effective fermentations at industrial scale might soon represent the turning point in fighting deficiencies in macro and micronutrients and in limiting the food industry environmental impact through production of stable and easy‐to‐delivery foods.

Overall, fermentation is a sustainable, efficient and low‐cost way to provide highly valuable, stable, safe and nutritional sources to vulnerable populations in low‐income countries, thus preventing malnutrition. This approach can further help to fulfil the United Nations Sustainable Development Goals (SDG) 2 and 3, which aim to end hunger and improve global health, respectively.

## FERMENTED FOODS FROM THE WORLD, A TAXONOMIC CHARACTERISATION

Each area of the world has its own indigenous FFs. The wide differentiation in raw materials and production technologies employed is linked with the availability of local food sources and with the cultural and religious heritage of the populations. Therefore, fermented meats are far less widespread in Far East regions (e.g., Japan and Korea), which most frequently ferment rice, sprouts and soy, whereas fermentation of milk and cheese production mostly developed in areas where the pastoral practices were more common in ancient times, such as India and European countries (Tamang et al., 2020). For the same reason, fermented fish are traditionally consumed by populations living in coastal regions who generally rely on fishing, such as Scandinavian countries (Tamang et al., 2016), while sorghum‐, maize‐ and wheat‐based FFs are typical from Africa (Tamang et al., 2020). All of these foods host diverse microbial communities, for which differences and similarities remain undescribed.

In order to depict a map of worldwide FF microbiota, we screened the available literature. Only studies using 16S rRNA gene sequencing were included. The studies were further filtered considering the availability of raw fastq sequences and metadata for each sample. Furthermore, we only included samples that were not spoiled. According to these criteria, 72 studies were selected, with a total of 2013 samples (Table S1). Data were downloaded and analysed through the QIIME 2 pipeline (Bolyen et al., 2019; q2cli version 2020.11.1). More specifically, raw reads from each BioProject were denoised independently using dada2 (options “‐‐p‐chimera‐method pooled,” “‐‐p‐pooling‐method pseudo,” “‐‐p‐min‐fold‐parent‐over‐abundance 10” and “‐‐p‐max‐ee 2”), producing Amplicon Sequence Variants (ASVs). Therefore, the ASVs were mapped against the Greengenes 13_8 database (McDonald et al., 2012) using the command “qiime feature‐classifier classify‐consensus‐vsearch,” and taxonomic information was used to independently collapse the ASVs tables at species level. Finally, all the collapsed abundance tables were merged to perform statistical comparisons.

**FIGURE 1:**
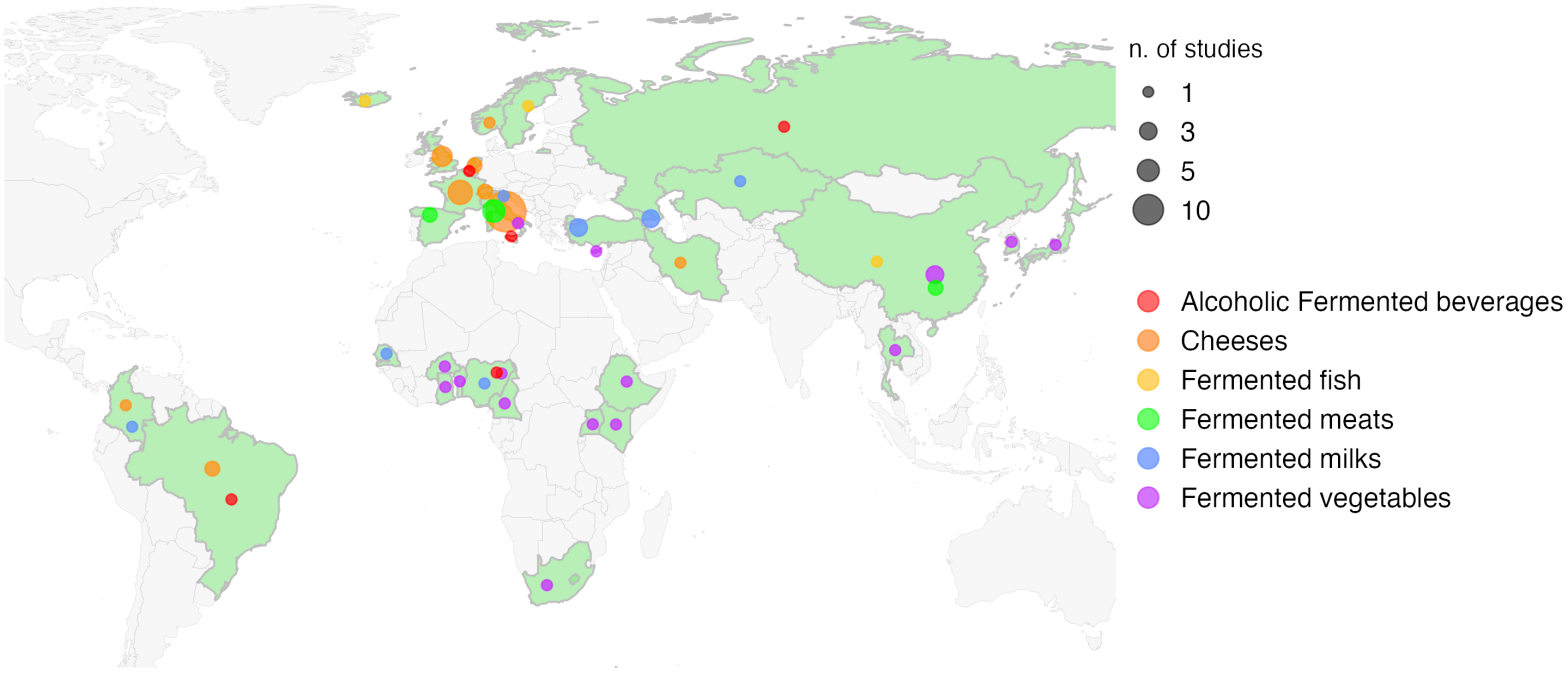
World map showing the number of studies included in the metataxonomic analysis focusing on traditional fermented foods from different countries (highlighted in green). Bubbles are colour‐coded according to the fermented food type. Bubble size is proportional to the number of different studies found in literature for each FF type.

Overall, the most studied FFs are cheeses from European countries (mainly Italy, France and United Kingdom), with a total of 33 studies and 1158 samples. However, European fermented meats (sausages and salami, *n* = 7 studies and 101 samples) were also frequently studied (Figure 1).

Interestingly, the microbiota of Asian and African fermented vegetables and cereals were also frequently studied. The high humidity and temperature of tropical and subtropical countries in Asia and Africa can cause perishable products to spoil quickly. To overcome this, ancient populations fermented vegetables and cereals to extend their quality and ensure safety. These traditional methods have been passed down, leading to a variety of FFs still consumed in Asia and Africa (Swain et al., 2014). Moreover, 5 studies focused on fermented milk from Caucasian area (i.e., kefir and yogurt), although the microbial composition of local fermented milk from Nigeria, Senegal, Italy and Colombia was also characterised by single studies (Figure 1). Fermented fish and alcoholic fermented beverages were generally less characterised, with only 3 and 5 studies, respectively.

**FIGURE 2:**
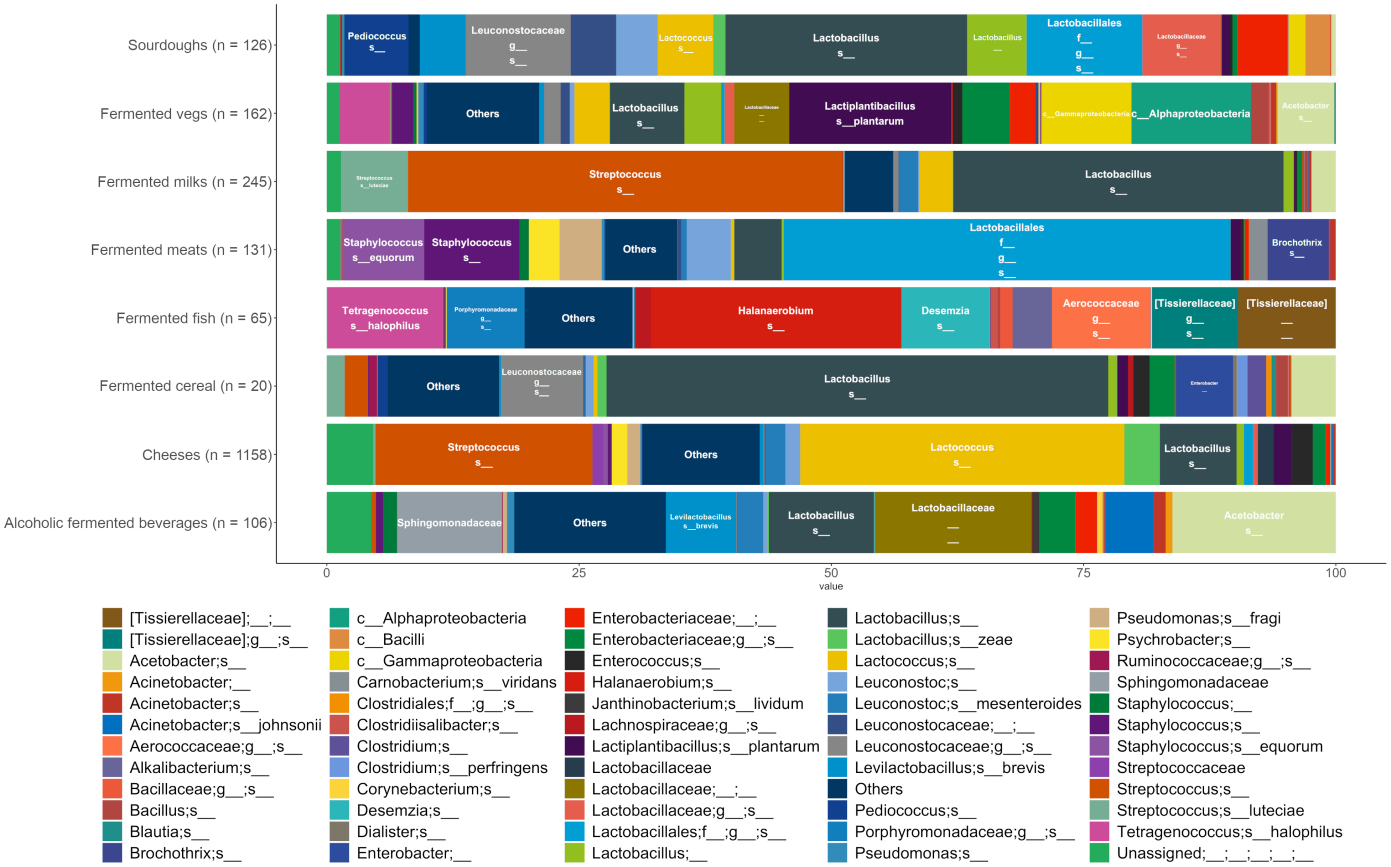
Bar plot showing, for each fermented food category, the average relative abundance of the taxa occurring in >25% of the group samples with an abundance >0.5%. All taxa with abundance <0.5% were included in “Others.”

For each type of FF, Figure 2 shows the average composition of its microbiota (occurring in >25% of the samples of the group with a relative abundance >0.5%). Interestingly, *Lactobacillus* spp. (that includes also the genera arising from its recent reclassification, Zheng et al., 2020) is highly prevalent in all the groups except fermented fish, with an intragroup average abundance ranging from 4.68% (in fermented meats) to 32.7% (in fermented milk). The result is not surprising: *Lactobacillus* spp. have a pivotal role in food fermentations, given the massive phenotypic and genotypic diversity, which led to the selection of multiple species, well adapted to different food niches (Widyastuti et al., 2021).

However, apart from *Lactobacillus* spp., several group‐specific prevalent taxa can be identified. Indeed, *Tetragenococcus halophilus*, *Halanaerobium* sp. and members of the *Tissierellaceae* and *Aerococcaceae* families are exclusively found in fermented fish. The occurrence of the halophilic *T. halophilus* and *Halanaerobium* sp. (Kim et al., 2022; Lipus et al., 2017) can be explained by the addition of salt to fermented fish (e.g., a Chinese fish sauce and the Swedish Surströmming). Interestingly, a *T. halophilus* strain isolated from a Korean fermented soybean has been identified as a potential probiotic starter culture for salty fermented foods (Kim et al., 2022). Similarly, *Tissierellaceae* have been reported as dominant in hákarl, traditional Icelandic fermented shark also included in this review (Osimani et al., 2019, Table S1).

*Lactiplantibacillus plantarum* (formerly *Lactobacillus plantarum*) showed high prevalence and average relative abundance in fermented vegetables (that includes pickles, olives and fermented soybeans; Figure 2). Research reports this species as being dominant in spontaneously fermented vegetables (Filannino et al., 2016; Owade et al., 2021; Xiong et al., 2012; Yang et al., 2014), and a recent review summarised the protechnological role of *L. plantarum* during fermentation of vegetables, which included enhancement of flavour, competition against pathogens in silage and boost of nutritional properties (Yilmaz et al., 2022). Hence, some *L. plantarum* strains have been characterised in depth to produce fine‐tuned starter cultures widely used nowadays (Li et al., 2022; Zhang et al., 2022).

Fermented vegetables included in this review were also dominated by *Acetobacter* sp., being present in high abundance in cocoa beans, with an average relative abundance >25% (Figure 2). *Acetobacter* spp. perform acetic fermentation (see par. 1), described as a key process in chocolate production as it triggers a cascade of reactions resulting in the release of chocolate aroma precursors (Soumahoro et al., 2020). In addition, *Acetobacter* was also found in alcoholic fermented beverages, where it may cause spoilage. Indeed, when alcoholic concentration is <10%, *Acetobacter* spp. can oxidise ethanol into acetic acid, developing off‐flavours and ropiness in wines, beers and cider (Bisson & Walker, 2015; Kubizniaková et al., 2021). However, in some specific brewery technologies, a well‐controlled acetic acid fermentation by *Acetobacter* spp. might lead to the production of sour beers (Bouchez & De Vuyst, 2022).

**FIGURE 3:**
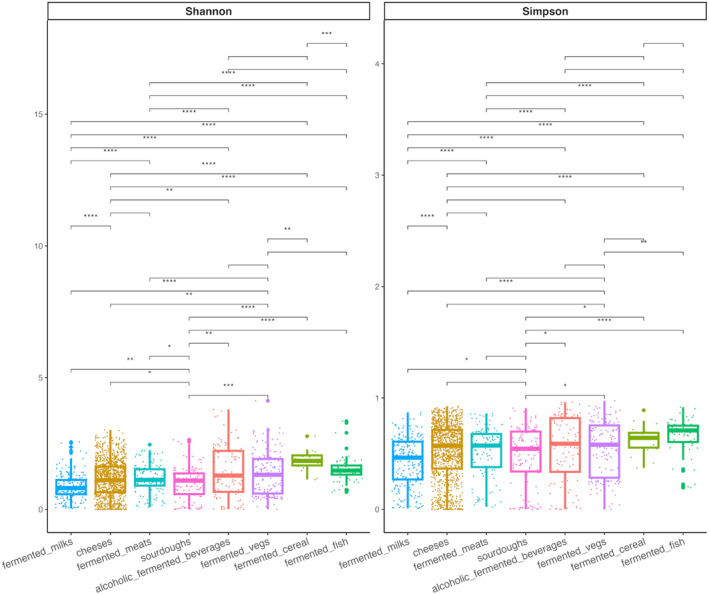
Box plot showing the Shannon's and Simpson's alpha diversity indices in each fermented food group. The means of the indices are compared across the groups using paired Wilcoxon's rank‐sum test. *p < 0.05; **p < 0.01; ***p < 0.001; ****p < 0.0001.

Overall, fermented dairy products and meats harbour the lowest biodiversity (Figure 3).

This suggests that these products host a selected microbiota and is consistent with observations that these foods are mainly composed of *Lactobacillus*, *Lactococcus* and *Streptococcus* in dairy products and *Lactobacillaceae*, *Staphylococcaceae* and *Brochotrix* in fermented meats. While the main genera occurring in dairy products are involved in fermentation and are recognised as “Generally Recognized As Safe” (GRAS) and/or the “Qualified Presumption of Safety” (QPS) status in the United States and UE, respectively, some of the microorganisms present in fermented meats might cause spoilage (e.g., *Brochothrix*, linked with off‐odours and discoloration; Stanborough et al., 2017) or raise safety concerns. Generally, fermented meats are populated by coagulase‐negative staphylococci (CNS), differing from the coagulase‐positive staphylococci (e.g., *Staphylococcus aureus*) that include human pathogens (Lee et al., 2018). The CNS group includes *S. carnosum*, *S. xylosus* and *S. equorum*, a bacterium firstly isolated from a healthy horse which enhances the colour of fermented meats through reduction of nitrates to nitrites. In addition, reports suggest that this species might influence the sensorial profile of sausages and cheeses through the production of butan‐3‐diol, acetoin and diacetyl (Irlinger et al., 2012; Lee et al., 2018; Stavropoulou et al., 2018), generating pleasant or off‐flavours depending on the ratio between these molecules and other volatile organic compounds (VOCs). However, it was also proved that *S. equorum* strains isolated from FFs may become resistant to multiple antibiotics through acquisition of mobile genetic elements (Heo et al., 2022).

As the samples were not reported as spoiled in the original publications, the high abundance of *Brochothrix* sp. and *Pseudomonas fragi*, coming from the raw meat used and considered as the main meat spoilers (Sequino et al., 2022), might be counteracted by *Lactobacillaceae* (Barcenilla et al., 2022; Favaro & Todorov, 2017; Zhang et al., 2018).

Despite the limited taxonomic resolution of 16S rRNA sequencing‐based approaches (Gupta et al., 2019), we provided valuable information about the average taxonomic composition and microbial diversity of several FF categories. Although not exhaustive, these results show that LAB are widespread in different FFs from all over the world, highlighting their key role in defining the sensory characteristics and protecting FFs from spoilage and pathogenic microorganisms. Besides LAB, each FF group showed its own microbial community that resulted from the composition of the raw materials (Leech et al., 2020), from the production environment (De Filippis, Valentino, et al., 2021) and from the selection occurring during the specific fermentation, as a result of the technological parameters applied.

## FERMENTATION BOOSTS THE NUTRITIONAL VALUE OF FOODS

Fermentation can also boost nutritional properties of the food matrices by either enhancing mineral bioavailability or reducing the concentration of toxic molecules such as mycotoxins (Adebo et al., 2019). Yogurt is one of the most studied FF, and research data suggest that fermentation of milk into yogurt increases the concentration and the bioavailability of several essential minerals, such as calcium and potassium (Hadjimbei et al., 2022). In addition, LAB may synthetise group B vitamins—mainly B2 and B12—which are essential vitamins for humans (Fabian et al., 2008).

Similarly, *Lactobacillus kefiranofaciens* produces kefiran, a peculiar exopolysaccharide (EPS) of kefir lacking in milk, which shows both prebiotic and protechnological properties (Moradi & Kalanpour, 2019; Vieira et al., 2021).

The enrichment in nutrients as a consequence of fermentation has been also observed in different food matrices. Indeed, Verni et al. (2019) showed that fermentation of faba bean flour with a *Lactiplantibacillus plantarum* strain improved the release of essential free amino acids and their bioactive peptide derivatives such as γ‐aminobutyric acid (GABA; Verni et al., 2019), a neurotransmitter often linked with stress reduction and benefiting brain health (see Section: “Fermented foods and their effects on human health through the gut microbiome: evidence from clinical trials”). Moreover, Jiang et al. (2021) observed similar results after fermentation of corn gluten meal and wheat bran with *Lb. delbrueckii* subsp. *bulgaricus* and *Lb. acidophilus*. Generally, improvements in the amino acid composition of FFs can be linked to LAB, which show a wide range of exo‐ and endopeptidases needed to release the free amino acids necessary for their growth (Kieliszek et al., 2021).

In addition, a decrease in antinutritional compounds, allergens or other molecules that can cause gastrointestinal discomfort may occur after food fermentation. Indeed, several LAB harbour the β‐galactosidase (lactase) enzyme, which hydrolyses lactose into glucose and galactose. Consequently, fermented milk products like yogurt and long‐ripened cheeses contain little or no lactose, which makes them suitable for lactose‐intolerant people (Melini et al., 2019). Moreover, the action of LAB proteases and peptidases on milk proteins such as alpha‐lacto‐albumin and beta‐lacto‐globulin enhances the release of short bioactive peptides with radical scavenging, metal chelating and peroxidation‐preventing activities, protecting human cells from oxidative stress (Pessione & Cirrincione, 2016). Several *Lb. helveticus* strains produce lactotripeptides (e.g., Val‐Pro‐Pro and Ile‐Pro‐Pro) with antihypertensive activities (Raveschot et al., 2018), and it is also known that *Lactobacillus* spp. proteolytic systems lead to the release of peptides enhancing the activity of anti‐inflammatory cytokines involved in allergies and macrophage phagocytosis. Importantly, the biosynthesis of most of the bioactive peptides can be linked to *Lactobacillus* spp. (and all genera arising from its reclassification, Zheng et al., 2020), which generally encode more peptidases, proteases and transport systems than other LAB, such as *Lactococcus* spp. (Pessione & Cirrincione, 2016). Also, these proteolytic systems provide LAB with the ability to release free amino acids (Nielsen et al., 2022). Indeed, all these reports highlighted the role of fermentation in improving the nutritional value of foods, possibly contributing to prevent malnutrition and contribute to well‐being, as auspicated in the SDG3.

Fermentation may also reduce antinutritional compounds naturally occurring in the raw materials. As an example, cereals and legumes naturally have high concentration of phytates, derivatives of phytic acids that are negatively charged and considered antinutritional compounds as they are able to bind positively charged minerals (e.g., zinc, calcium and iron), thus chelating them and reducing intestinal absorption upon consumption. It has been shown that fermentation results in phytate reduction, and several phytase‐producing strains from both bacterial and fungal species have been identified (Greppi et al., 2015; Mohammadi‐Kouchesfahani et al., 2019). Similarly, food fermentation of vegetable raw materials facilitated by *Lactobacillaceae* can denature cyanogenic glycosides, a class of toxic compounds present in >2000 plant species which causes acute intoxication (Bolarinwa et al., 2016).

Furthermore, several studies highlighted the ability of *Lactobacillus* and *Bifidobacterium* spp. probiotic strains to denature the indigested gliadin‐derived peptides, responsible of the immune response in celiac disease, thus suggesting an exciting possibility of exploiting fermentation to produce gluten‐free products (Norouzbeigi et al., 2020).

## FERMENTED FOODS: AN EXPLOITABLE SOURCE OF POTENTIALLY PROBIOTIC STRAINS

According to the ISAPP, probiotics are “live microorganisms which confer a health benefit on the host when administered in adequate amounts” (Hill et al., 2014). As such, microbial strains have to satisfy several criteria to be considered probiotic. First of all, a probiotic candidate must be safe for its intended use to be administered to humans (Sanders et al., 2010), and the safety of an isolate has to be proven at strain‐level (European Food Safety Authority, 2007). However, some taxonomic groups are not of concern from a pathogenicity perspective as research and a long history of use has established their safety. These microbial groups have gained the aforementioned GRAS and/or QPS status, which provides a simplified pathway for the application of isolates from widely recognised safe species. Secondly, beneficial activities of putative probiotics must be proven by multiple randomised double‐blind placebo‐controlled trials, which represent the gold standard in intervention studies (Misra, 2012). Moreover, high‐quality systematic reviews can provide evidence of a causal effect between probiotic administration and health effects (Hill et al., 2014). Finally, beneficial outcomes on human health are dose‐dependent, therefore probiotics should reach the body site where the positive action is exerted (e.g., the gut) in adequate amounts.

Given these assumptions, FFs represent an optimal source of potentially probiotic strains, although fermentation does not necessarily lead to the selection of probiotics, therefore the terms “fermented food” and “probiotic” cannot be used interchangeably (Marco et al., 2021; Vinderola et al., 2023). Firstly, most of the confirmed probiotic strains belong to genera often involved in food fermentations such as the former *Lactobacillus* and *Lactococcus* (Lavefve et al., 2019). Indeed, several species from these genera have been assigned QPS and GRAS status, given the long history of safe consumption and commensalism with humans. Streptococci represent an exception: Although *S. thermophilus* is frequently involved in food fermentations and probiotics have been characterised from this species, the genus also includes some human pathogens (Plummer et al., 2021). However, phylogenetic studies suggest that *S. thermophilus* followed a separate evolutionary path from other *Streptococcus* species, leading to the loss of virulence genes (De Filippis et al., 2020).

Microbial strains and whole communities might reach loads up to 10^8^ CFU/g in some traditional FFs (Leeuwendaal et al., 2022; Rezac et al., 2018). This might ease the technological process of isolating autochthonous potentially probiotic strains with an enhanced growth rate. Once isolated and characterised, these strains might be inoculated into food matrices with chemical and physical properties that mimic those of the isolation source. In such a way, the FF might become a carrier of a new probiotic (Papadopoulou et al., 2023), which might in turn reach the human gut in adequate amounts to have a beneficial impact. This strategy, also known as “probiotication,” is widely used for the production of dairy products (Mojikon et al., 2022), but recent advances aim to extend it to the production of plant‐based FFs (Di Cagno et al., 2013; Kumar et al., 2015; Mustafa et al., 2019).

After ingestion, one desirable trait of probiotic strains is to tolerate adverse conditions encountered through the gastrointestinal tract that might pose a limit to the survival of an adequate number of cells. For example, microorganisms targeting the human gut should tolerate acid environments and high concentrations of bile salts and digestive enzymes (e.g., lipases, amylases and nucleases) while transiting through the stomach and duodenum, respectively. Interestingly, Wang et al. (2018) isolated >30 LAB strains resistant to low pH and high bile concentrations from spontaneously fermented vegetables and meats, similarly to Meena et al. (2022), who found 6 candidate probiotic strains of *Lb. delbrueckii* subsp. *bulgaricus* and *Lacticaseibacillus rhamnosus* in fermented cereals from India. To explain this, Di Cagno et al. (2013) suggested that autochthonous and potentially probiotic strains from fermented and non‐fermented vegetables might naturally show resistance to stressful environmental conditions thanks to some traits shared between their isolation source and the gut, such as the high acidity and concentration of antinutritional components. Interestingly, some structures produced by LAB such as exopolysaccharides (EPS) can be both an important component for the texture of certain fermented foods (Han et al., 2016) and a tool to protect cells from environmental stress (De Filippis et al., 2020).

Another desirable trait of probiotic strains is adherence to the host gut epithelium, which promotes its persistence in the gut after the consumption. Several cell structures can equip probiotics with this ability; membrane receptors like lipoteichoic acids (LTA) and mucin‐binding proteins spread in some LAB genera enhance the bonds between the microorganism and the glycosylated part of the gut mucin (Monteagudo‐Mera et al., 2019). Overall, these traits are highly prevalent within the species commonly found in FFs (De Filippis et al., 2020; Garcia‐Gonzalez et al., 2018), although defining the ability of probiotics to establish in the gut is still challenging (Roselli et al., 2021). However, recent evidence suggests that either a permanent or transitory establishment of microorganisms introduced with FFs in the gut might occur. For example, a large‐scale analysis of LAB genomes reconstructed from gut and FF metagenomes highlighted that for several LAB species (e.g., *S. thermophilus* and *Lc. lactis*), genomes from the two environments have a high genomic similarity, suggesting that the intake of FFs might be the primary source of these species in the human gut (Pasolli et al., 2020). This hypothesis was further supported by focusing on the LAB prevalence in different cohorts, which showed that several *Leuconostocaceae* and *Weissellaceae* taxa, commonly found in traditional Asiatic fermented vegetables and cereals, were more frequently detected in the gut metagenomes of non‐Westernised cohorts (e.g., Far East populations).

Several factors might contribute to defining the extent of establishment in the gut. Firstly, strain‐specific metabolic adaptive traits can be involved in engraftment in the human gut. Indeed, it was shown that *Lactiplantibacillus plantarum* 299v is able to adapt its metabolic potential towards carbohydrate hydrolysation and production of compounds involved in adherence (Derrien & van Hylckama Vlieg, 2015). Furthermore, the personalised gut microbiome response can influence the establishment of food‐introduced microbes. Maldonado‐Gómez et al. (2016) observed that the engraftment of a *Bifidobacterium bifidum* strain was strongly dependent on the gut microbiome composition of the individuals and favoured by the presence of phylogenetically related taxa in the gut. However, the mechanisms underlying this interaction are mostly not yet explained. Lastly, the persistence of a strain in the gut is also strongly linked with the intake. Indeed, it was estimated that the consumption of 10^10^ cells might alter the gut microbiota composition temporarily, potentially modulating its immune and neuroendocrine activities in the short term (Derrien & van Hylckama Vlieg, 2015).

Notably, even though the human gut still remains the main source of commercially available probiotic strains (i.e., those strains with beneficial effects supported by clinical trials; Hill et al., 2014), some probiotics have been isolated from FFs. Indeed, *Lb. helveticus* Rosell‐52, a strain that can contribute to ameliorate immune functions and modulate the gut–brain axis (Murina et al., 2021), was firstly isolated from dairy‐fermented products (McFarland, 2015), while *Lacticaseibacillus rhamnosus* HN001 originates from cheese (Ceapa et al., 2015). Besides bacteria, some yeast strains commonly found in FFs have been screened for probiotic activities. For example, *Saccharomyces cerevisiae* var. *boulardii* was first isolated from lychee and mangosteen, and is frequently detected in kombucha and kefir (Ansari et al., 2023). Collectively, these recent outcomes suggest that FFs might represent an underexplored source of highly competitive, stress‐tolerant and easy‐to‐propagate strains with potential beneficial outcomes on human health, therefore further efforts should focus on the screening of FF microbial communities for novel probiotics.

## FERMENTED FOODS AND THEIR EFFECTS ON HUMAN HEALTH THROUGH THE GUT MICROBIOME: EVIDENCE FROM CLINICAL TRIALS

**FIGURE 4:**
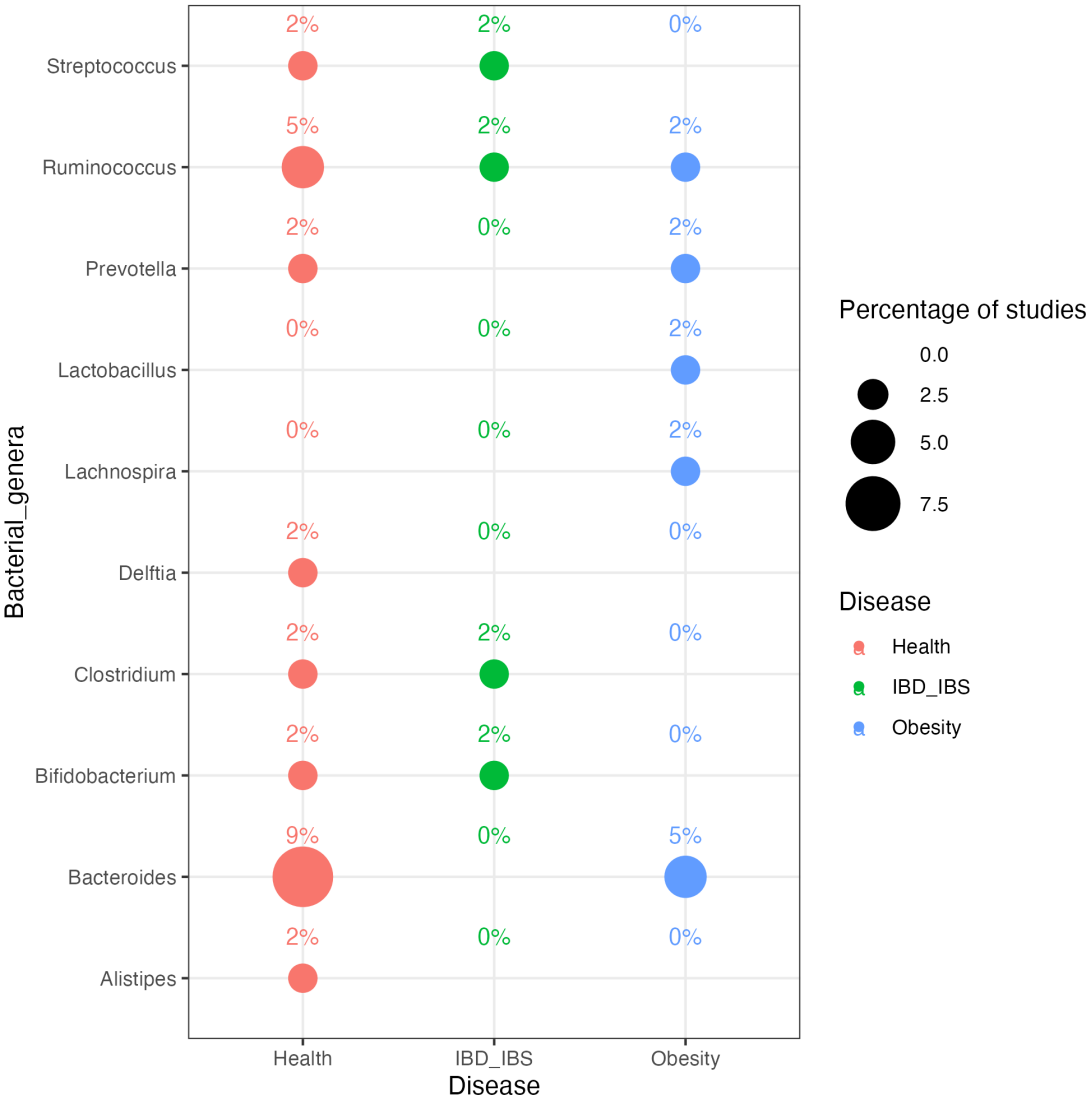
Bubble plot showing the percentage of studies (from those summarised in Table 3) in which microbial genera increase after an intervention with different FFs. Bubble size is proportional to the number of different studies reporting the same association. The group labelled as “Health” corresponds to those intervention studies where the population was described as “Healthy.” The total number of intervention studies reported in this analysis is 44.

The gut microbiome, a diverse and dynamic community of microorganisms that inhabit the human gastrointestinal tract, has a crucial function in host physiology and state of health (De Filippis et al., 2018). For instance, a disequilibrium in the gut microbiome composition may lead to abnormalities in the immune system, resulting in diseases such as obesity, diabetes, allergies and inflammatory bowel disease (Lin & Zhang, 2017). Among the variety of factors, food is thought to be one of the most important variables in modulating the gut microbiome throughout life (Dominianni et al., 2015; Wang et al., 2020). Indeed, FFs that are rich in living and/or inactivated microorganisms and nutritional components released from fermentation, may modulate the gut microbiome (Zhang et al., 2016). In order to investigate how consuming FFs may have a crucial role on human health, we performed a search using the MeSH (Medical Subject Headings) terms in the PubMed database (https://pubmed.ncbi.nlm.nih.gov/) with the query reported in Appendix S1. This search resulted in about 59,000 results when filtered by year (from 1980 to 2023) in March 2023. Abstracts were screened to include observational and intervention studies that used qPCR, DGGE, 16S rRNA and shotgun sequencing to analyse the gut microbiome and evaluated health outcomes; although it is clear that gut microbiome analyses and health outcome investigations are not always conducted within the same study. Interventions consisting of only probiotics were also not considered in this review. As reported above, the fermentation process might improve nutritional value of the FFs beyond mere nourishment. This hypothesis is supported by human clinical investigations on FFs, as reported in Tables 2 and 3, summarising observational and intervention studies. In particular, Table 2 summarises observational studies on the effects of diets containing different FFs on gut microbiome and associated health benefits. In this case, the studies vary in the whole diet composition of the participants, as well as the study design, the control group used for comparison, the participant characteristics and health‐related parameters measured. Smith‐Brown et al. (2016) investigated the effect of a diet rich in dairy products, including cheese and yogurt, on the gut microbiome of a cohort of 37 normal‐weight children, comparing them with a plant‐based diet group. The study reports changes in the relative abundance of several bacterial taxa, including *Streptococcus thermophilus*, *Erysipelatoclostridium ramosum* and *Faecalibacterium prausnitzii*; however, no health effect was monitored. Taylor et al. (2020) evaluated the effect of the habitual consumption of a diet rich in various plant‐based FFs on the gut microbiome, compared with a group of non‐consumers. The study reports differences in the relative abundance of several bacterial taxa in the gut microbiome, including several taxa with a potential positive effect on human health, such as *Bacteroides*, *Dorea*, *Prevotella* and *Faecalibacterium prausnitzii*, which were higher in the consumer group. These taxa have been suggested as potential next‐generation probiotics (NGPs), that are “microbial taxa that conform to the traditional definition of probiotics, but do not have an history of use for health promotion.” (De Filippis et al., 2022). Anyway, the metabolome of FFs consumers was also analysed in the same study (Taylor et al., 2020), finding an enrichment in conjugated linoleic acid, a molecule supposed to be health‐promoting (Marangoni et al., 2020). Another observational study by González et al. (2019) demonstrated an increase of taxa suggested as NGPs upon FFs consumption. In particular, *Akkermansia muciniphila*, a promising NGP (Zhang et al., 2019), increased in consumers of yogurt (González et al., 2019). Moreover, in the same study, yogurt consumption was positively correlated with healthier metabolic profile in terms of lower inflammation and serum lipid peroxidation. In Table 3, several studies on the effects of dietary intervention with FFs on the gut microbiome and health outcomes are summarised. The studies vary in the FF matrices analysed and in the daily quantity consumed, as well as in the study design, participant characteristics and health outcome targeted. These studies suggest that FFs, such as kefir, kombucha, yogurt and fermented milk, may have a positive effect on gut microbiome composition, reducing the abundance of detrimental bacteria and increasing the abundance of beneficial taxa. A visualisation of microbial genera that increase after intervention, based on the results from studies reported in Table 3, is shown in Figure 4.

**TABLE 2:** Observational studies investigating the effects of fermented food consumption on human health.

Diet and fermented food matrix	Brief diet description	Study design	Control group	Participants	Participant characteristics	Gut microbiome analysis method	Variation in gut microbiome detected	Health outcome targeted	Health outcome achieved	Reference
Lacto‐fermented vegetables	Balanced diet including a 2‐year‐long daily consumption of lacto‐fermented vegetables	Cohort, comparison of consumer groups versus non‐consumers	Non‐consumers	47	Male (29.79%), adults (20–50 years), normal‐weight	16S rRNA and ITS2	Bacteria: ↑ *Leuconostoc mesenteroides*, *Parabacteroides distasonis*, *Lachnospira*, *Ruminococcaceae*, *Coproccocus*, *Blautia*, *Clostridiales*, *Coprobacillus*, *Alphaproteobacteria* Fungi: ↑ *Rhodotorula mucilaginosa*, *Penicillium*, *Starmerella*, *Cryptococcus laurentii* and *Vishniacozyma carnescens*	None	‐	Guse et al. (2023)
Dairy products	Balanced diet including cheese, yogurt and dairy milk among fermented foods high‐intake	Cohort, comparison of groups with high versus low intake of FFs	Plant‐based diet	37	Male (56.76%), children (2–3 years), normal‐weight	16S rRNA	↑ *Streptococcus salivarius* subsp. *thermophilus*, *Erysipelatoclostridium ramosum*, *Lachnoclostridium* ↓ *Faecalibacterium prausnitzii*, *Fusicatenibacter*, *Alistipes*, *Bacteroides*, *Parabacteroides*	None	‐	Smith‐Brown et al. (2016)
Dairy products	Balanced diets including yogurt, natural yogurt, sweetened yogurt, cheese, matured/semi‐matured cheese, fresh cheese, fermented milk	Cross‐sectional, comparison of consumer groups versus non‐consumers	Non‐consumers	130	Male (29.23%), adults (58.18 ± 17.10 years), normal‐weight and overweight	qPCR	↑ *Akkermansia muciniphila* ↓ *Bacteroides*	Cardiometabolic Disease and metabolic syndrome	Yes	González et al. (2019)
Fermented milk	Balanced with a fermented milk high‐intake	Case–control, comparison of groups affected by ASD versus not affected	Children not affected by ASD	46	Male (100%), children (4–9 years), normal‐weight and obese	16S rRNA	↑ *Clostridia*, *Lactobacillus*, *Blautia*, *Anaerostipes* and *Fusicatenibacter*	Autism spectrum disorder (ASD)	Yes	Tomova et al. (2020)
Fermented products	Diet including yogurt, wine, sauerkraut, pickled vegetables, kombucha, kimchi and beer, aside from fermented plants	Cohort, comparison of consumer groups versus non‐consumers	Non‐consumers	115	Male (52.60%), adults (19–70 years), normal‐weight	16S rRNA	↑ *Bacteroides*, *Pseudomonas*, *Dorea*, *Lachnospiraceae*, *Prevotella*, *Alistipes putredinis*, *Oscillospira*, *Enterobacteriaceae*, *Fusobacterium*, *Actinomyces*, *Achromobacter*, *Clostridium clostridioforme*, *Faecalibacterium prausnitzii*, *Bacteroides uniformis*, *Clostridiales* and *Delftia*	None	‐	Taylor et al. (2020)
Yogurt and Probiotic Fermented Milk (PFM)	Not available	Cohort, comparison of consumer groups versus non‐consumers	Non‐consumers	272	Male (49.26%), adults (25–50 years), normal‐weight and overweight	16S rRNA	↑ *Bifidobacterium*	None	‐	Redondo‐Useros et al. (2019)
Asian and African diet	Lacto‐vegetarian diet	Cross‐sectional, comparison between Asian versus African diet	Control‐based Asian/African	100	Male (0%), adults (16–35 years), normal‐weight	16S rRNA	↑ *Bifidobacterium*, *Succinivibrio* and *Escherichia*‐*Shigella* in Asian population ↑ *Ruminococcus*, *Lachnospiraceae*, *Succinivibrio* and *Faecalibacterium* in African population	None	‐	Tang et al. (2019)
Kazakh nomads diet	High‐fat, high‐meat and low‐vegetable diet	Cross‐sectional, comparison among interindividual variations	Control‐based interindividual variations	29	Male (37.93%), adults (6–84 years), normal‐weight and overweight	16S rRNA	↑ *Bifidobacterium* and *Collinsella aerofaciens*	None	‐	Li et al. (2019)
Rural Saudi Arabian diet	Diet characterised by six plant‐based fermented foods, called Lohoh in the local language	Cross‐sectional, comparison between rural versus urban Arabian diet	Urban Saudi Arabian diet	28	Male (92.86%), adults (>18 years), weight not reported	16S rRNA	↑ *Verrucomicrobaeota*, *Acetobacter*, *Mycoplasma*, *Treponema berlinense* and *Treponema succinifaciens*	None	‐	Angelakis et al. (2016)
Sakura diet	Not available	Cohort, comparison among interindividual and intraindividual variations	Control‐based interindividual and intraindividual variations	10	Male (50.00%), adults (men, 37.2 years; women, 38.2 years) normal‐weight	16S rRNA	↑ *Bifidobacterium*	None	‐	Hisada et al. (2015)

**TABLE 3:** Intervention studies investigating the effects of fermented food consumption on human health.

Diet and fermented food matrix	Daily quantity of fermented food	Study design	Control group	Participants	Participant characteristics	Length of intervention	Wash out	Gut microbiome analysis method	Variation in gut microbiome detected	Health outcome targeted	Health outcome achieved	Reference
High‐fermented foods diet (cottage cheese, kefir, kombucha, vegetable brine drinks, vegetables, yogurt, other foods and drinks)	6 servings	Randomised, prospective	High‐fibre diet (fruits, grains, legumes, nuts/seeds, vegetables, meat, dairy and other)	36	Male (30.56%), adults (52 ± 11 years), normal‐weight	17 weeks	No	16S rRNA and Shotgun	↑ *Ruminococcaceae* and *Streptococcaceae* ↓ *Lachnospira*	Inflammation	Yes	Wastyk et al. (2021)
Fermented milk	80 g	Cross‐over trial	No intervention	35	Male (60.00%), children (7–10 years), normal‐weight and overweight	4 weeks	Yes	16S rRNA	↑ *Bacteroides ovatus*, *Lachnospira* and *Ruminococcus*	Obesity	No	Joseph et al. (2020)
Fermented milk	100 g	Before/after trial	Control‐based before/after	21	Male (33.33%), adults (18–25 years), normal‐weight	2 weeks	Yes	16S rRNA	None	None	‐	Khine et al. (2019)
Fermented milk	100/300 g	Randomised, double‐blind, placebo‐controlled trial	Acidified milk	96	Male (45.83%), adults (18–55 years), normal‐weight	4 weeks	Yes	16S rRNA and Shotgun	None	None	‐	Alvarez et al. (2020)
Fermented milk	110 g	Randomised, double‐blind, placebo‐controlled trial	Pasteurised acidified milk	25	Male (36.00%), adults (treated, 36.9 ± 6.9 years; control, 36.5 ± 6.1 years), normal‐weight	10 weeks	No	16S rRNA	↑ *Collinsella*, *Lactobacillus*, *Blautia* and *Ruminococcus* ↓ *Bacteroides*, *Parabacteroides*, *Prevotella* and *Oscillospira*	Cedar pollinosis	Yes	Harata et al. (2017)
Fermented milk	250 g	Randomised, double‐blind, parallel and controlled trial	Non‐fermented milk product	106	Male (35.80%), adults (18–65 years), normal‐weight	2 weeks	Yes	16S rRNA	None	Irritable bowel syndrome	No	Le Nevé et al. (2019)
Fermented milk	250 g	Randomised, double‐blind, parallel and placebo‐controlled trial	Acidified milk	28	Male (0%), adults (20–69 years), weight not reported	4 weeks	Yes	Shotgun	↑ *Clostridiales* ↓ *Bilophila wadsworthia*	Irritable bowel syndrome	Yes	Veiga et al., 2014
Fermented milk	180 g	Double‐blind, randomise, placebo‐controlled, multicentric trial	Heat‐treated fermented milk	30	Gender not reported, adults (18–65 years), weight not reported	4 weeks	Yes	16S rRNA and qPCR	↑ *Streptococcus thermophilus* (short term, both treatment and control)	Irritable bowel syndrome	No	Bogovič Matijašić et al. (2016)
Fermented milk	140 g	Before/after trial	Control‐based before/after	6	Male (0%), adults (20–24 years), normal‐weight	3 weeks	Yes	16S rRNA	↑ *Bacteroidaceae* and *Prevotellaceae* ↓ *Ruminococcaceae* and *Lachnospiraceae*	None	‐	Unno et al. (2015)
Fermented milk	600 mL	Randomised, double‐blind and placebo‐controlled trial	Placebo	18	Male (100%), adults (21.6 ± 0.8 years), normal‐weight	2 days	No	Not detected	‐	Muscle soreness	Yes	Iwasa et al. (2013)
Fermented milk	125 g	Randomised, controlled, parallel trial	Non‐fermented milk and no intervention	36	Male (0%), adults (18–55 years), normal‐weight	4 weeks	No	16S rRNA	None	Brain intrinsic activity or emotional attention	Yes	Tillisch et al. (2013)
Fermented milk	80 mL	Randomised, placebo‐controlled, double‐blind trial	Placebo	72	Male (26.39%), (85 years), normal‐weight	6 months	No	qPCR	↑ *Bifidobacterium*, *Lactobacillus* ↓ *Clostridium difficile*, *C. perfringens*, *Enterobacteriaceae*, *Staphylococcus* and *Pseudomonas*	Infection control, bowel movement normalcy	Yes	Nagata et al. (2016)
Yogurt	125 g	Cross‐over trial, double blind	Pasteurised yogurt	79	Male (40.51%), adults (mean 23.6 years), normal‐weight	2 weeks	Yes	DGGE and qPCR	LAB and *C. perfringens* ↓ *Bacteroides*	None	‐	García‐Albiach et al. (2008)
Yogurt	500 g	Before/after trial	Control‐based before/after	20	Male (20.00%), adults (30 ± 5 years), normal‐weight	2 weeks	Yes	DGGE and qPCR	↑ LAB and *Clostridium perfringens* ↓ *Bacteroides*, *Prevotella*, *Porphyromonas*	None	‐	Vázquez et al. (2013)
Yogurt	220 g	Randomised, controlled trial	Milk	92	Male (0%), adults (milk, 51.2 ± 10.2 years; yogurt, 48.9 ± 10.5 years), obese	24 weeks	Yes	16S rRNA	↑ *Phascolarctobacterium* ↓ *Bacillaeota*, *Clostridiales*, *Blautia*, *Eubacterium ventriosum*, *Erysipelotrichaceae*, *Ruminococcus*, *Pseudobutyrivibrio*, *Dialister*	Metabolic syndrome	Yes	Chen et al. (2019)
Yogurt	400 g	Randomised, double‐blind, cross‐over trial	Acidified milk	14	Male (100%), adults (18–40 years), normal‐weight	2 weeks	Yes	16S rRNA	None	Postprandial inflammation	No	Burton et al. (2017)
Yogurt	250 g	Before/after trial	Control‐based before/after	135	Male (31.11%), adults (18–40 years), normal‐weight	30 days	No	16S rRNA	↑ *Bifidobacterium*, *Streptococcus*, *Actinobacteraeota* and *Erysipelotrichaceae*	None	‐	Volokh et al. (2019)
Yogurt	250 g	Open label trial, longitudinal over pregnancy and after delivery	No intervention	56	Male (0%), adults (18–40 years), pregnant	88 ± 31 days	No	16S rRNA	Mother's microbiota: no effects Newborns: ↑ *Bifidobacterium* ↓ *Enterobacteriaceae*	Undernourishment	Mother's microbiota: no Newborn faeces: yes	Bisanz et al. (2015)
Kefir	180 mL	Randomised, parallel and controlled trial	Milk	22	Male (27.27%), adults (18–65 years), normal‐weight and overweight	12 weeks	No	16S rRNA	↑ *Actinobacteria*	Metabolic syndrome	Yes	Bellikci‐Koyu et al. (2019)
Kefir	500 mL	Non‐randomised, uncontrolled intervention study	None	20	Male (40%), adults (27–78 years), normal‐weight	4 weeks	No	Not detected	‐	Functional constipation	Yes	Turan et al. (2015)
Kefir	500 mL	Randomised controlled trial, double‐blind	Milk	82	Male (56.10%), children and adults (12–46 years), normal‐weight	2 weeks	No	Not detected	‐	Dyspepsia and *H. pylori* infection	Yes	Bekar et al. (2011)
Kefir	508 mL (plain kefir); 519 g (raspberry‐flavoured kefir)	Randomised control trial, cross‐over	Low‐fat cow milk; plain yogurt; flavoured yogurt	15	Male (53.33%), adults (20–34 years), normal‐weight	5 days	No	Not detected	‐	Lactose malabsorption	Yes	Hertzler and Clancy (2003)
Kefir	75–150 mL	Randomised control trial, double‐blind	Heat‐treated kefir	125	Male (51.20%), children (1–5 years), normal‐weight	2 weeks	No	Not detected	‐	Antibiotic‐associated diarrhoea	No	Merenstein et al. (2009)
Kefir	400 mL	Randomised control trial	No intervention	45	Male (51.11%), adults (19–68 years), normal‐weight	4 weeks	No	qPCR	None	Irritable bowel disease	Yes	Yilmaz et al. (2020)
Kefir	1600 mg (supplemented with 1500 mg of CaCO_3_)	Randomised, parallel, controlled, double‐blind trial	Unfermented raw milk	40	Male (43.80%), adults (control group, 67.94 ± 8.37 years; kefir group 64.08 ± 14.51 years), normal‐weight	6 months	No	Not detected	‐	Osteoporosis	Yes	Tu et al. (2015)
Parmesan	45 g	Before/after trial	Parmesan with milk	20	‐	2 weeks	Yes	16S rRNA and Shotgun	None	None	‐	Milani et al. (2019)
Cured meats and cheeses	Ad libitum	Cross‐over trial	Plant‐based diet	20	Male (60%), adults (21–33 years), normal‐weight	15 days	Yes	16S rRNA and ITS	↑ *Alistipes*, *Bilophila* and *Bacteroides* ↓ *Roseburia*, *Eubacterium rectale* and *Ruminococcus bromii*	None	‐	David et al. (2014)
Chungkookjang (Korean fermented soy product)	35 g	Randomised, placebo‐controlled, cross‐over trial	Placebo pills	120	Male (60%), adults (19–29 years), normal‐weight	12 weeks	Yes	Not detected	‐	Obesity	Yes	Byun et al. (2016)
Fermented soy product supplemented with isoflavones	200 mL	Randomised, placebo‐controlled, double‐blind trial	Unfermented soy product	49	Male (100%), adults (37–57 years), normal‐weight	42 days	No	Not detected	‐	Cardiometabolic disease	Yes	Cardoso Umbelino Cavallini et al. (2016)
Kochujang (Korean fermented sauce)	34.5 g	Randomised, placebo‐controlled, double‐blind trial	Placebo pills	30	Male (43.33%), adults (19–55 years), normal‐weight	12 weeks	No	Not detected	‐	Hyperlipidemia	Yes	Lim et al. (2015)
Kimchi	180 g	Randomised, parallel and controlled trial	Unfermented kimchi	23	Male (0%), adults (30–60 years), obese	8 weeks	No	16S rRNA	↑ *Prevotella* and *Bacteroides* ↓ *Blautia*	Metabolic syndrome	Yes	Han et al. (2015)
Kimchi	300 g	Randomised, controlled, cross‐over trial	Unfermented kimchi	21	Male (33.3%), adults (31–65 years), normal‐weight and overweight	8 weeks	Yes	Not detected	‐	Prediabetes	Yes	An et al. (2013)
Kimchi	210 g	Randomised, parallel and controlled trial	Low kimchi consumption (15 g/day)	100	Male (50%), adults (low‐intake group, 22.5 ± 3.1 years; high‐intake group 23.0 ± 2.8 years), normal‐weight	1 week	No	Not detected	‐	Cardiometabolic disease	Yes	Choi et al. (2013)
Kimchi	100 g	Randomised, open‐labelled, prospective, and controlled trial	No intervention	43	Gender not reported, adults (over 20 years), normal‐weight	4 weeks	Yes	Not detected	‐	Immune system	No	Lee et al. (2014)
Kimchi	210 g	Randomised, parallel and controlled trial	Functional kimchi (210 g/day)	28	Male (64.29%), adults (standard kimchi, 22.6 ± 2.2 years functional kimchi, 24.1 ± 4.8 years), normal‐weight	4 weeks	No	16S rRNA	Kimchi: ↑ *Bacteroidaeota* and *Actinobacteraeota* ↓ *Bacillaeota*, *Alphaproteobacteraeota* and *Tenericutes. Bifidobacterium adolescentis* Functional kimchi: ↑ *Bacteroidaeota* and *Alphaproteobacteraeota. Bifidobacterium adolescentis* ↓ *Bacillaeota* and *Actinobacteraeota*	Metabolic syndrome and irritable bowel syndrome	Yes	Kim and Park (2018)
Psychobiotic diet (high in prebiotic fibres, legumes and FFs)	6–8 servings/day (prebiotic fibres); 5–8 servings/day (grains); 3–4 servings/week (legumes), 2–3 servings/day (FFs)	Single‐blind, randomised, controlled trial	No intervention	45	Male (37.78%), adults (18–59 years), normal‐weight	4 weeks	No	Shotgun	None	Perceived stress	Yes	Berding et al. (2023)
Sauerkraut (unpasteurised and containing LAB)	75 g	Randomised, double‐blind controlled trial	Pasteurised sauerkraut	58	Male (62.50%), adults (21–59 years), normal‐weight	6 weeks	No	16S rRNA	↑ *Clostridiales*, *Lachnospiraceae*, *Ruminococcaceae Faecalibacterium*, *Eubacterium* and *Bacteroides*	Irritable bowel syndrome	Yes	Nielsen et al. (2018)
Sourdough	145 g	Randomised cross‐over trial	White wheat bread	20	Male (45%), adults (18–70 years), normal‐weight	1 week	Yes	16S rRNA and Shotgun	White wheat bread: ↑ *Eubacterium ventriosum* and *Anaerostipes* spp.	Cardiometabolic disease	‐	Korem et al. (2017)
Sourdough	2 sourdough croissants	Randomised, double‐blind, cross‐over, controlled trial	Traditional yeast croissants	17	Male (48%), adults (18–40 years), normal‐weight	Single study day	Yes	Not detected	‐	Gastric emptying and gastrointestinal fermentation symptoms	Yes	Polese et al. (2018)
Sourdough	6–10 slices	Randomised cross‐over trial	Wheat bread enriched with fermented rye bran	7	Male (57.14%), adults (38–61 years), normal‐weight	4 weeks	No	Not detected	‐	Gastrointestinal symptoms	Yes	Raninen et al. (2017)
Sourdough	7–8 slices	Randomised, double‐blinded, cross‐over trial	Traditional sourdough rye bread	87	Male (8.7%), adults (21–64 years), normal‐weight	4 weeks	Yes	Not detected	‐	Irritable bowel syndrome	Yes	Laatikainen et al. (2016)
Sourdough	6 slices	Randomised controlled trial, double‐blinded	Yeast‐fermented wheat bread	26	Male (4%), adults (21–64 years), normal‐weight	7 days	No	Not detected	‐	Irritable bowel syndrome	No	Laatikainen et al. (2017)
Sourdough	200 g	Non‐randomise, uncontrolled trial	None	8	Gender not reported, children (8–17 years), normal‐weight	60 days	No	Not detected	‐	Coeliac disease	Yes	Di Cagno et al. (2010)
Sourdough	200 g	Randomised controlled trial	Traditional wheat bread	20	Male (30%), children and adults (12–24 years), normal‐weight	3 days	No	Not detected	‐	Coeliac disease	Yes	Mandile et al. (2017)

**FIGURE 5:**
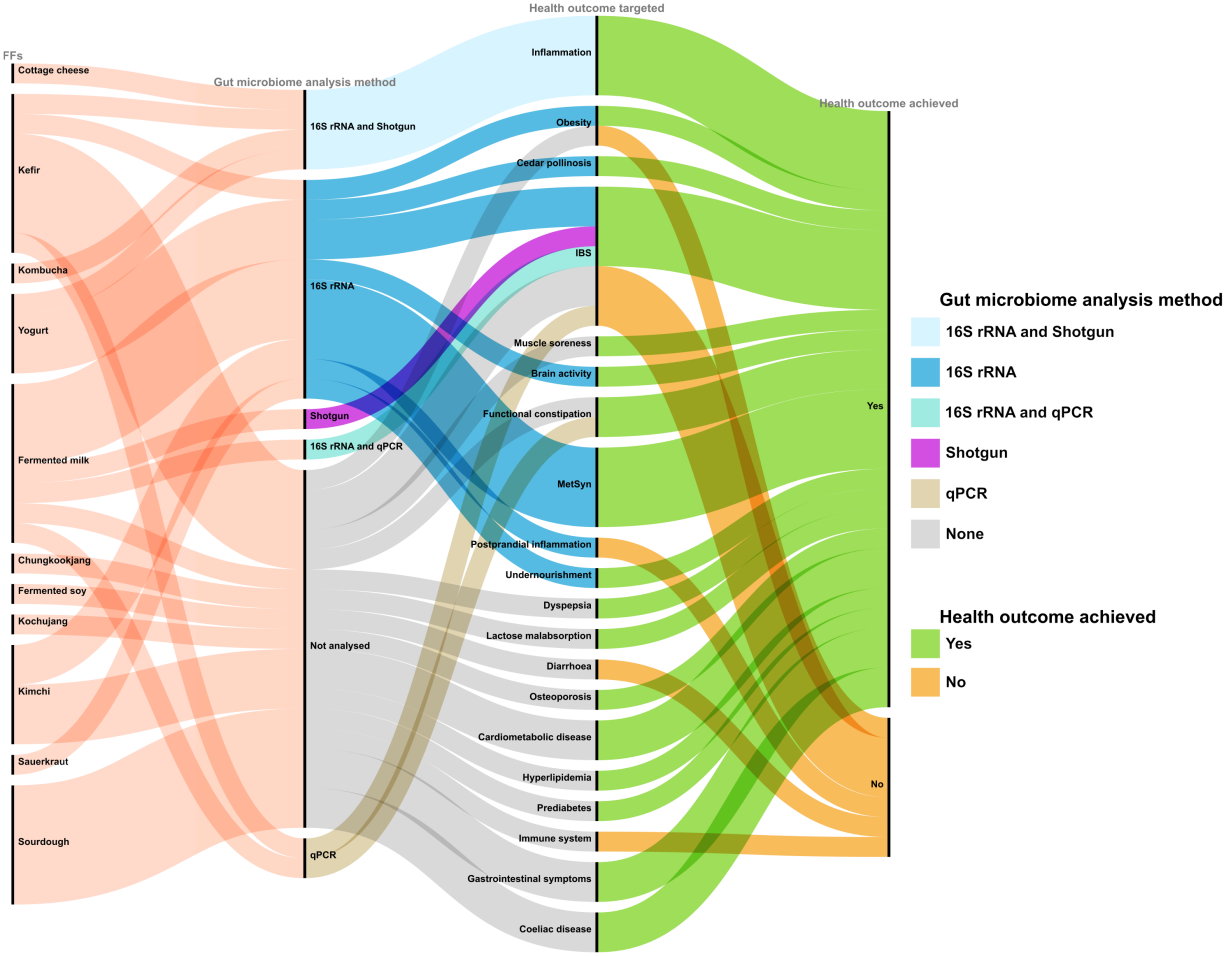
Alluvial diagram highlighting the correlation among FFs, microbiome analysis methods, health outcomes targeted and achieved in trials reported in Table 3.

The variation in gut microbiome detected was often specific to the type and quantity of FFs consumed. For instance, a recent trial compared the effects of different microbiota‐targeted dietary interventions (i.e., high‐FF and high‐fibre diets) on gut microbiota and immune response (Wastyk et al., 2021). The goal of this study was to increase the number of FFs servings per day from <1 to at least 6 in the high‐FF group. FFs included yogurt, kefir, fermented cottage cheese, kombucha, vegetable brine drinks and fermented vegetables. A high‐fibre diet, on the other hand, was characterised by an average fibre intake of 45.1 ± 10.7 g per day. The authors showed that several taxa were shared between FFs and gut microbiota upon intervention, with the greatest overlap occurring in the early phase of the intervention. Moreover, the investigation revealed compelling evidence of different effects between the two dietary interventions on the diversity of the gut microbiota. Specifically, a high‐FF diet boosted a significant and noteworthy increase in microbial diversity. Conversely, a high‐fibre diet did not result in substantial alterations in the overall abundance of species within the gut microbial community. Notably, an increase in Bacillota was observed, distributed across the *Lachnospiraceae*, *Ruminococcaceae* and *Streptococcaceae* families in the high‐FF group. However, results from clinical trials present in the literature are sometimes discordant, and not all the studies showed a significant improvement in health outcomes, such as inflammation, obesity and irritable bowel syndrome (IBS), suggesting that the relationship among FFs, gut microbiome and health is complex and may depend on various factors. In fact, Joseph et al. (2020) reported an increase in *Bacteroides ovatus*, *Lachnospira* and *Ruminococcus* in the gut microbiome of participants following an intervention of 4 weeks with a fermented milk. The study targeted obesity as the health outcome but did not find a significant effect. The relative abundance of *Lachnospira* spp., a bacterial genus well‐known for producing short‐chain fatty acids (SCFAs) and associated with a variety of health outcomes, increases significantly in FFs trials targeting obese participants. Several studies reported an increase in *Prevotella* upon FFs consumption. *Prevotella* spp. has been mooted as a diet and lifestyle biomarker, owing to its extensive presence in varied worldwide populations (Tett et al., 2021). Indeed, *Prevotella* species have a unique metabolite profile linked with the breakdown and digestion of complex dietary polysaccharides, indicating that dietary fibre fermentation is occurring (Precup & Vodnar, 2019). Dietary patterns associated with *Prevotella* spp. are largely plant‐based, with high‐fibre content, and this taxon is often associated with rural and non‐Westernised dietary patterns (Medawar et al., 2019). Furthermore, vegetarian, vegan and Mediterranean diets are linked to increased *Prevotella* levels (De Filippis et al., 2016), although species and strain‐level differences may subsist (De Filippis et al., 2019; Tett et al., 2021). We also identified several studies consistently reporting an increase in *Ruminococcus* spp. in different target populations during trials with FFs. *Ruminococcus* spp. was often reported as correlated with both IBS and inflammatory bowel disease (IBD) (Bolte et al., 2021). In particular, *Ruminococcus gnavus* showed enrichment in people with IBD (Zhai et al., 2023) and specific *R. gnavus* strains are linked with inflammation associated with food allergies (De Filippis, Paparo, et al., 2021; De Filippis, Valentino, et al., 2021). However, other *Ruminococcus* species (e.g., *R. bromii* and *R. albus*) have been associated with a positive influence on human health (Scott et al., 2015; Ze et al., 2012). Furthermore, according to Chen et al. (2019), traditionally fermented yogurt was more efficient than milk in decreasing insulin resistance in obese women with metabolic syndrome, potentially through a modulation of lipid metabolism, inflammation, oxidative stress and shifting the composition of the gut microbiota. In another intervention study focused on the consumption of fresh cabbages or fermented kimchi (Han et al., 2015) in obese women, fermented kimchi intake yielded considerable and positive changes in the gut microbiota, including increases in *Bifidobacterium* and *Lactobacillus*, while reducing the harmful *Clostridium perfringens*. Berding et al. (2023) highlighted that the consumption of FFs is also related to mental health. An intervention with a diet rich in FFs and fibre (defined psychobiotic diet, PD) led to a decrease in perceived stress. Moreover, although no significant change in gut microbiome composition was detected, the intervention with the PD boosted the changes in a total of 53 distinct faecal, plasmatic and urinary metabolites compared with the control diet. Thus, a diet rich in FFs may be beneficial for the neuronal networks, suggesting that consuming FFs can affect also mood and brain activity (Kok & Hutkins, 2018; Selhub et al., 2014). Some commensal and mutualistic bacteria may have a key role in treating stress‐related disorders such as anxiety and depression that are closely linked to functional bowel disorders (Bravo et al., 2011). Alterations in the receptor expression of the main central nervous system inhibitory neurotransmitter, γ‐aminobutyric acid (GABA), contribute to the pathogenesis of anxiety and depression (Pehrson & Sanchez, 2015). FFs may alleviate symptoms of depression and anxiety (Aslam et al., 2020; Selhub et al., 2014). A combination between advances in shotgun metagenomic sequencing and new clinical trials should make it possible to characterise the microbial and molecular relationships between FFs and human health in greater depth (Walsh et al., 2023). If consuming FFs can alter and enrich the human gut microbiome in the long term, FFs may help to prevent the significant loss of gut microbial diversity that has been linked with several chronic diseases typical of Western lifestyle (Segata, 2015). However, some of the studies found in the literature highlight inconsistent results (Table 3 and Figure 5). This may be explained by multiple variables, such as different target populations, quantity of FFs examined, and the diversity of microbial species and/or strains contained in FFs (Raoult & Henrissat, 2014), as well as gut microbiome interindividual differences, that may influence the response to dietary interventions (De Filippis et al., 2018). In fact, it was previously shown that gut microbiome interindividual variability, local dietary habits and genetic background are all components of an intricate interplay between dietary intakes and health outcomes (Chen et al., 2022). Indeed, these factors may contribute to the observed discrepancies within the examined literature. As a result, it is critical to evaluate these factors in order to get more accurate conclusions on the possible health effects of FFs. In several cases, intervention studies do not specifically target alterations in the gut microbiome following the consumption of FFs (Figure 5). Furthermore, it is essential to underscore that a significant portion of the analysed studies showed in Figure 5, and aiming to characterise the gut microbiome, employed the 16S rRNA sequencing method or even more outdated molecular analysis (i.e., DGGE and qPCR), that do not target the entire genomic content of a sample and do not allow to reach the strain‐level characterisation. As a consequence, a more in‐depth characterisation of the gut microbiome would be necessary in order to explore FFs‐gut microbiome relationships, since a strain‐level diversity in the gut exists (De Filippis et al., 2019, 2020; Tett et al., 2019) and this may influence the impact of dietary or drug treatments (Vinderola et al., 2023). In addition, most of the available interventions are conducted on yogurt and other dairy products. Thus, expansion of randomised controlled trials to other FFs is needed, in order to address these knowledge gaps. In addition, in‐depth focus on the molecular mechanisms behind the beneficial effects of FFs on human health is also necessary. Overall, we provide a useful summary of different studies on the effects of various FFs on the gut microbiome composition and associated health outcomes.

Specifically, although it has been speculated that consuming FFs may introduce new microorganisms in the human gut, there is still a lack of evidence in tracking microbial strains that pass from FFs to human gut microbiome (Marco et al., 2017). In such a way, the application of shotgun metagenomic sequencing for strain tracking provides an opportunity for future studies that want to further investigate the delivery of microbes and the associations with human health (Quince et al., 2017). All this knowledge may support the development of personalised and tailored interventions targeting the gut microbiome to promote human health (Virgin & Todd, 2011). Anyway, while numerous association and correlation studies have linked the ingestion of live microbes through FFs to various health benefits, a comprehensive understanding of the cause‐and‐effect relationship between their consumption and specific health outcomes remains a field that warrants further investigation and clinical trials. Indeed, Marco et al. (2022) classified different foods according to the quantity of live microbes per gram in high (mainly FFs and raw vegetables), medium and low live microbe content. Using this classification, screened the American National Health and Nutrition Examination Survey (NHANES) database was screened to identify associations between the live microbe intake and several diseases (Hill et al., 2023). They highlighted that the quantity of live microbes in the diet is negatively correlated with systolic blood pressure, inflammation, body mass index, insulin and triglycerides levels. Therefore, they suggested that defining a minimal daily intake dose of live microorganisms is crucial to minimise the risk of chronic diseases and improve population health. Clearly further research and properly designed clinical trials are needed to better understand the mechanisms explaining the link of FF live microbes with specific health outcomes, focusing on determining the optimal type and quantity of FFs that can positively impact human health and if this effect may be modulated by the gut microbiome. Additionally, long‐term interventions with larger populations are needed to confirm the findings present in literature. However, nowadays, ultra‐processed foods pervade Westernised diets, which lead to a decreased diversity of the gut microbiome and can have significant health implications, such as chronic inflammation (Sonnenburg & Sonnenburg, 2019). The human gut microbiome interacts not only with microorganisms, but also with their leftover compounds. Indeed, metabolites like acetic, butyric and lactic acids, EPS, and proteins may improve the immunity associated with the mucosal membrane of the gastrointestinal tract, lowering inflammation (Mathur et al., 2020). Through this mechanism, FFs may protect against gastrointestinal tract disorders, food allergies, bacterial or viral infections, Crohn's disease and more (Parvez et al., 2006). Even though there have been relatively few human dietary intervention studies involving FFs, most of the evidence points to the positive effects of eating FFs on human health, particularly in the treatment of metabolic diseases, the management of weight, mood and mental health, and the reduction of overall mortality (Hill et al., 2023; Marco et al., 2017). Indeed, FF consumption may represent a valuable and low‐cost alternative to promote well‐being and prevent non‐communicable diseases, in line with the objectives of the SDG3.

## CONCLUSIONS AND FUTURE PERSPECTIVES

Overall, FFs are widespread and the multiple combinations of matrices and fermentation technologies concur to define ecological niches harbouring microbial communities different in composition and functions, which are still partially unexplored. In this Review, we focused on the complexity of FF microbiota, highlighting that traditional products harbour highly selected microbial communities. Furthermore, we provided an up‐to‐date review of the literature focusing on the effects of FF consumption on the human health, including the most recent observational/intervention studies, which supports the idea that human health might take advantage from FF consumption. Indeed, they may actively modulate both the composition and the functionality of the human gut microbiome, leading to the selection of health‐related species or functions. In addition, since microorganisms are exposed to similar environmental pressures in some FFs and in the human gut, we suggested that traditional FFs might be an exciting and unexplored source of novel probiotic strains, able to survive to the gastrointestinal passage. Unfortunately, the direct transfer of microorganisms from FFs to the human gut remains not proved yet and the possible underlying mechanisms explaining the link between FFs and human health are still to be fully uncovered. However, the field of FFs microbiome research is on an exciting wave with promising advancements, and Next‐Generation Sequencing technologies coupled with machine learning approaches will help researchers to unravel the molecular networks existing between microbial communities in FFs and human health outcomes, as well as to identify new probiotic strains potentially boosting human health.

As we explore the molecular interactions within FFs, refined dietary recommendations are expected, considering both health benefits and ecological impact. The convergence of cutting‐edge research, technological innovations and dietary guidelines place FFs not just as culinary delights but as sustainable contributors to human well‐being.

## AUTHOR CONTRIBUTIONS

**Vincenzo Valentino:** Formal analysis (equal); writing – original draft (equal). **Raffaele Magliulo:** Formal analysis (equal); writing – original draft (equal). **Dominic Farsi:** Writing – review and editing (equal). **Paul D. Cotter:** Funding acquisition (equal); writing – review and editing (equal). **Orla O'Sullivan:** Funding acquisition (equal); writing – review and editing (equal). **Danilo Ercolini:** Conceptualization (equal); funding acquisition (equal); writing – review and editing (equal). **Francesca De Filippis:** Conceptualization (equal); funding acquisition (equal); writing – original draft (equal).

## FUNDING INFORMATION

This work was partially supported by the Italian Ministry of Foreign Affairs and International Cooperation, with a grant to the project FOODMICROHERITAGE—“Quality and authenticity protection of artisanal fermented foods through the characterization and conservation of their microbial and genetic heritage” (VN21GR09) and by the project DOMINO—“Harnessing the microbial potential of fermented foods for healthy and sustainable food systems.” This project has received funding from the European Union's Horizon Europe research and innovation programme under grant agreement No 101060218. The manuscript reflects only the authors' views, and the European Commission is not responsible for any use that may be made of the information it contains. The work was also supported by the National Recovery and Resilience Plan (NRRP), Mission 4, Component 2, Investment 1.4 “Strengthening of research structures and creation of R&D ‘national champions’ on some Key Enabling Technologies” ‐ Call for tender No. 3138 of 16 December 2021, rectified by Decree n. 3175 of 18 December 2021 of Italian Ministry of University and Research funded by the European Union—NextGenerationEU; Project code CN_00000033, “National Biodiversity Future Center—NBFC.”

## CONFLICT OF INTEREST STATEMENT

The authors declare no conflicts of interest.

## Supporting information


Table S1

Appendix S1
