# Supplementary material for: Fermented foods, their microbiome and its potential in boosting human health
Source: Microb Biotechnol. 2024 Feb 23;17(2):e14428. doi: 10.1111/1751-7915.14428 (PMC10886436; doi:10.1111/1751-7915.14428)
Supplement: Supplementary file 2 — Appendix S1 [file MBT2-17-e14428-s002.html]

Fermented foods, their microbiome and its potential in boosting human health


# Fermented foods, their microbiome and its potential in boosting human health

#### PubMed query

## Why we decided to use MeSH terms:

Harnessing the might of MeSH terms in your PubMed quest can be
transformative. It streamlines the often daunting task of ferreting out
relevant articles with remarkable ease and precision. To illustrate this
power, consider a PubMed search for the MeSH term “fermented foods.”
What unfolds is PubMed’s impressive ability to summon forth articles
seamlessly, even if the words “fermented” and “foods” remain absent from
the article’s title or abstract.

```
(("fermented foods"[MeSH Terms] OR "fermented foods"[All Fields]) 
OR ("fermented plants"[MeSH Terms] OR "fermented plants"[All Fields]) 
OR ("dairy products"[MeSH Terms] OR "dairy products"[All Fields]) 
OR ("dairies"[MeSH Terms] OR "dairies"[All Fields]) 
OR ("fermented milk"[MeSH Terms] OR "fermented milk"[All Fields]) 
OR ("cheese"[MeSH Terms] OR "cheese"[All Fields]) 
OR ("cottage cheese"[MeSH Terms] OR "cottage cheese"[All Fields]) 
OR ("vegetable brine drinks"[MeSH Terms] OR "vegetable brine drinks"[All Fields]) 
OR ("parmesan"[MeSH Terms] OR "parmesan"[All Fields]) 
OR ("fermented meats"[MeSH Terms] OR "fermented meats"[All Fields]) 
OR ("salami"[MeSH Terms] OR "salami"[All Fields]) 
OR ("kimchi"[MeSH Terms] OR "kimchi"[All Fields]) 
OR ("yogurt"[MeSH Terms] OR "yogurt"[All Fields]) 
OR ("yoghurt"[MeSH Terms] OR "yoghurt"[All Fields]) 
OR ("yogourt"[MeSH Terms] OR "yogourt"[All Fields]) 
OR ("yoghourt"[MeSH Terms] OR "yoghourt"[All Fields]) 
OR ("kefir"[MeSH Terms] OR "kefir"[All Fields]) 
OR ("kombucha"[MeSH Terms] OR "kombucha"[All Fields]) 
OR ("sauerkraut"[MeSH Terms] OR "sauerkraut"[All Fields]) 
OR ("pickled vegetables"[MeSH Terms] OR "pickled vegetables"[All Fields]) 
OR ("beer"[MeSH Terms] OR "beer"[All Fields]) 
OR ("wine"[MeSH Terms] OR "wine"[All Fields])) 
OR ("century egg"[MeSH Terms] OR "century egg"[All Fields])) 

AND 

(("health"[MeSH Terms] OR "health"[All Fields]) 
OR ("inflammation"[MeSH Terms] OR "inflammation"[All Fields]) 
OR ("irritable bowel syndrome"[MeSH Terms] OR "irritable bowel syndrome"[All Fields]) 
OR ("irritable bowel disease"[MeSH Terms] OR "irritable bowel disease"[All Fields]) 
OR ("IBS"[MeSH Terms] OR "IBS"[All Fields]) 
OR ("IBD"[MeSH Terms] OR "IBD"[All Fields]) 
OR ("obesity"[MeSH Terms] OR "obesity"[All Fields]) 
OR ("metabolic syndrome"[MeSH Terms] OR "metabolic syndrome"[All Fields]) 
OR ("diabetes"[MeSH Terms] OR "diabetes"[All Fields]) 
OR ("postprandial glucose"[MeSH Terms] OR "postprandial glucose"[All Fields]) 
OR ("undernourishment"[MeSH Terms] OR "undernourishment"[All Fields]) 
OR ("cardiovascular disease"[MeSH Terms] OR "cardiovascular disease"[All Fields]) 
OR ("stroke"[MeSH Terms] OR "stroke"[All Fields]) OR 
("heart attack"[MeSH Terms] OR "heart attack"[All Fields]) 
OR ("immune system"[MeSH Terms] OR "immune system"[All Fields]))
```
